# Exogenous coenzyme Q10 modulates MMP-2 activity in MCF-7 cell line as a breast cancer cellular model

**DOI:** 10.1186/1475-2891-9-62

**Published:** 2010-11-30

**Authors:** Massih Bahar, Shahnaz Khaghani, Parvin Pasalar, Maliheh Paknejad, Mohammad Reza Khorramizadeh, Hossein Mirmiranpour, Siavash Gerayesh Nejad

**Affiliations:** 1 Department of Clinical Biochemistry, Tehran University of Medical Sciences, Faculty of Medicine, Tehran, Iran; 2 Department of Pathology, Tehran University of Medical Sciences, Faculty of Public Health, Tehran, Iran

## Abstract

**Background/Aims:**

Matrix Metalloproteinases 2 is a key molecule in cellular invasion and metastasis. Mitochondrial ROS has been established as a mediator of MMP activity. Coenzyme Q_10 _contributes to intracellular ROS regulation. Coenzyme Q_10 _beneficial effects on cancer are still in controversy but there are indications of Coenzyme Q_10 _complementing effect on tamoxifen receiving breast cancer patients.

**Methods:**

In this study we aimed to investigate the correlation of the effects of co-incubation of coenzyme Q10 and N-acetyl-L-cysteine (NAC) on intracellular H2O2 content and Matrix Metalloproteinase 2 (MMP-2) activity in MCF-7 cell line.

**Results and Discussion:**

Our experiment was designed to assess the effect in a time and dose related manner. Gelatin zymography and Flowcytometric measurement of H2O2 by 2'7',-dichlorofluorescin-diacetate probe were employed. The results showed that both coenzyme Q10 and N-acetyl-L-cysteine reduce MMP-2 activity along with the pro-oxidant capacity of the MCF-7 cell in a dose proportionate manner.

**Conclusions:**

Collectively, the present study highlights the significance of Coenzyme Q_10 _effect on the cell invasion/metastasis effecter molecules.

## Introduction

Matrix Metalloproteinases (MMPs) belong to a multigene family of enzymes that are mainly involved in physiological alterations of Extracellular Matrix (ECM). MMP-2 (Gelatinase A) and MMP-9 (Gelatinase B) are key enzymes in the degradation of ECM collagen, therefore ECM remodeling. MMPs are secreted in the form of inactive zymogens, which proceed to activation by various pathways. Both expression and activity of MMPs are regulated by diverse endogenous and exogenous stimuli (e.g. signals from cell-to-cell interaction and cellular microenvironment) [1].

The function of MMPs (especially MMP-2) is extensively studied because of their pivotal role in pathogenesis of certain diseases. MMP-2 has long been established as a marker in predicting phenotypes of tumor including growth, progression and metastasis as well as the dysregulated angiogenesis that is associated with these events. As a result, MMPs have come to represent important therapeutic and diagnostic targets for the therapy and diagnosis of human cancers in which their increase presages invasive phenotypes [2] and [3].

MMPs are expressed in form of latent MMPs (proMMP or zymogen) in response to exogenous signals, such as growth factors, cytokines, chemical agents like phorbolesters, physical stress, oncogenic transformation, cell -cell and cell-matrix interactions. ProMMPs are activated by disruption of the cysteine-zinc bond (switch) or by cleavage of the propeptide by proteinases such as plasmin, trypsin, kallikrein, chymase, and mast cell tryptase. Some latent MMPs can also be activated by another MMP. The disruption of the cysteine switch by different organic and inorganic compounds, like organomercurials, SH-reactive agents, reactive oxygen and detergents results in autocatalytic cleavage of the propeptide and a conformational change into catalytically active form [4].

As a consequence of the use of oxygen in aerobic respiration, oxygen radicals are naturally produced in all mammalian cells. Superoxide is generated within the mitochondria and is sequentially reduced to hydrogen peroxide (H_2_O_2_) and hydroxyl radicals. These radicals damage DNA, producing the mutations that initiate tumors and sustain progression.

Human tumor cell lines in vitro produce ROS at a far greater rate. Markers for the constitutive oxidative stress have been detected in samples from in vivo breast carcinomas and cell lines. 8-Hydroxy-2'-deoxyguanosine, one of the major oxidatively modified DNA base products, is almost ten times more prevalent in invasive ductal breast carcinoma cells than in normal control samples from the same patient [5]. The observed increase of ROS in transformed cells is not only the incendiary of further oxidative damage but also a mediator of certain pathological signal transduction pathways. Several important signal transduction pathways such as MAPK, PI3K, Rho-GTPase and Smads cascades, are known to mediate transcriptional regulation of metastasis-related genes such as integrins and MMPs [6].

As a consequence to the general knowledge of ROS implications in cancer, several studies have examined the effect of individual and combinational antioxidant dietary supplements on cancer outcome [7]. Despite equivocal conclusions in certain areas, there are supportive publications that antioxidant regiments would complement tamoxifen therapy in breast cancer patients in various aspects [8].

Enthusiasm for introducing new phytochemicals and supplements as potential therapeutic or preventive agents for cancer and other pathologic conditions has propelled researchers to study the behavior of key components of malignancy in the presence of these antioxidants. Therefore, evidence is rapidly mounting on the effect of various novel antioxidants on MMPs activity/expression in such studies [9] and [10]. However, the majority of recently introduced antioxidants are hydrophilic. This property delimits certain antioxidant applications, namely membrane passage and membrane protections.

Among four major groups of natural lipid-soluble antioxidants (carotenoids, tocopherols, estrogens and coenzyme Q) only Coenzyme Q is mostly present in reduced form. Coenzyme Q, in addition to membrane lipids, protects proteins and DNA due to its ubiquitous nature [11]. A lipid-soluble, endogenic, membrane-protective antioxidant, Coenzyme Q_10 _has been frequently employed in studies on vascular and atherosclerotic diseases. Consequently, its multiple roles in health and disease are still under vast examination and review [12]. Its multiple-reductive enzymatic mechanisms and presence in mitochondrial membrane endows Coenzyme Q_10 _an important place in the natural antioxidant defense system. Since oxidative stress is a predominating event in most pathological conditions, it is assumed that in those conditions, Coenzyme Q amount increases as an adaptive response to neutralize the pathogenic generated ROS. Moreover, in pathologic conditions, the majority of coenzyme Q is in reduced form, showing that the reductive enzymatic mechanism is not a limiting factor [13].

In addition to the properties mentioned above, recent findings in coenzyme Q_10 _capabilities to improve the survival of progressed cancer patients had made coenzyme Q_10 _a potential candidate in our study [14].

Several authors have focused on effects of coenzyme Q_10 _on breast cancer patients generally supporting the idea of ameliorating effects of coenzyme Q_10 _in the disease [15,16] and [17]. Alternatively, studies show that baseline plasma Coenzyme Q_10 _levels are a powerful and independent prognostic factor that can be used to estimate the risk for pancreatic and melanoma progression [18] and [19]. Coenzyme Q_10 _effect on MMPs has not been documented previously. Therefore, in this study we aim to elucidate the effect of exposure of a breast cancer cell line to exogenous Coenzyme Q_10 _on expression/activity of MMP in the microenvironment. To investigate this effect, we employed MCF-7, a renowned model of cancerous breast ductal epithelium [20].

Parallel to coenzyme Q_10_, we manipulated monothiol antioxidant content. It has been proven that supplementation of certain thiol antioxidants has inhibitory effect on MMP activity [21]. Therefore, we investigated the effects of alterations in monothiol antioxidant content by adding NAC and Buthionine Sulfoximine (BSO), a substantial competitive inhibitor of γ-glutamylcysteine synthetase (γ-GCS). BSO is a specific inhibitor of γ-GCS and is commonly employed to deplete the GSH content of a cell [22] and [23]. We assumed that this would also help us to obtain a better understanding of the regulatory effects of monothiol antioxidants (GSH and NAC) on MMP-2 gelatinolytic action in the MCF-7 breast cancer cell line. To our knowledge, there is a paucity of publication in which the effect of BSO on MMP-2 was investigated.

## Methods

### Cells and reagents

MCF-7 cell lines derived from pleural metastasis of a ductal human breast carcinoma were obtained from the Iranian National Cell Bank. DMEM, L-glutamine, FCS and antibiotics were purchased from Gibco. 2'7',-dichlorofluorescin-diacetate, Sodium bicarbonate, NAC, BSO and Coenzyme Q10 were from Sigma. The liquids were from Merck. After culturing HT1080 in a serum-free medium for 48 hours, we collected the supernatants and used them as a molecular weight marker of pro-MMP-2 and MMP-2 as described in literature [24].

### Preparation of the reagents

NAC was dissolved in DMEM in serial concentrations from 2 mM to 0.5 mM. Coenzyme Q_10 _was dissolved in 40° ethanol and added to DMEM in a sublethal concentration of ethanol in 1×, 0.5 ×s and 0.25 ×s of 122 μM concentrations. DMEM with similar concentrations of ethanol was prepared for the control wells.

### Preparation of MCF-7 samples

MCF-7 cells were cultured in DMEM supplemented with 10% heat-inactivated FCS, antibiotics and 2 mm L-glutamine at 37°C in a 7% CO_2_-humidified atmosphere. Cells were routinely passaged twice a week at a 1:10 split ratio. From the 48 h cultured cells, 2 × 10^5 ^cell were seeded into each well of a 24-well culture plate in the presence of 5% FCS, and incubated for 6, 15, 24 and 42 hours. By completion of the incubation time, the media over the cells were aspirated and underwent flowcytometric analysis. A portion of the media was kept in -70°C for further analysis.

### Fluorescent Measurement of H_2_O_2 _content

Hydrogen peroxide as a marker of cellular ROS production was examined by a method dependent on intracellular deacylation and oxidation of 2', 9'-dichlorodihydrofluorescein diacetate (DCHF-DA) to the fluorescent compound 2', 7'9-dichlorofluorescein (DCF). This probe is highly reactive with H_2_O_2 _and has been used in evaluating ROS generation in mammalian cells [25].

To assess the levels of intracellular ROS, flow cytometric analysis was performed using the oxidative-sensitive probe (DCFH-DA) as described [26]. Cells were incubated for 30 minutes at 37°C in the presence of 5 mM DCFH-DA. After incubation, the cells were transferred to an ice bath, and the formation of 2', 7'- dichlorofluorescein (DCF) was analyzed by flowcytometry using a Becton Dickinson Facscan with excitation and emission settings of 495 and 525 nm, respectively. Ten thousand viable cells from quadruple samples were analyzed.

### Gelatin Zymography

To assess the expression/activation of MMP-2, Sodium Dodecyl Sulfate PolyAcrylamide Gel Electrophoresis (SDS-PAGE) substrate-embedded enzymography (zymography) was carried out by a modification of the method [27]. Briefly, SDS-PAGE gels were prepared for mini-gels from 30:1 acrylamide/bis with the incorporation of gelatin (1 mg/ml) before casting. The gelatin gels were routinely 7.5% acrylamide. Various denatured but non-reduced samples and standards were then run into the gels at constant voltage of 150 V under non-reducing conditions. When the dye fronts reached a point approximately 0.5 cm from the bottom of the gels, the gels were removed and subjected to the following washing protocol: twice for 30 minutes each time in 50 mM Tris buffer (containing 1 mM Ca^2+ ^and 0.5 mM Zn^2+^) with 2.5% Triton X-100; once for 5 minutes in Tris buffer alone and finally overnight in Tris buffer with 1% Triton X-100 [28].

The molecular weight marker of pro-MMP-2 and MMP-2 and inhibitors were added to the overnight wash. Then the gels were stained with Coomassie Brilliant Blue 250-R. After destaining, zones of enzyme activity showed up as regions of negative staining. Relevant controls included samples incubated with EDTA (MMP inhibitors), PMSF (serine proteinase inhibitor), E64 (cysteine proteinase inhibitor) and pepstatin (aspartic proteinase inhibitor). HT1080 culture supernatant was used as the molecular weight marker of pro-MMP-2 and MMP-2 [29].

### Statistical analysis

Values were expressed as MMP-2 activity percentage of control samples, means and standard errors. For effectiveness and dose-response studies, differences among groups were evaluated with two-way analysis of variance (ANOVA). If the F-value was significant, groups were then compared at each dose by one-way analysis of variance (ANOVA) followed by Dunnett's C test (non-homogenous variances) and Schaffe (homogenous variances) Post Hoc analysis. Bivariate correlation one-tailed Spearman tests were performed for the determination of correlation between H2O2 vs. supplement concentration and H2O2 vs. MMP-2 activity. If a p value was less than 0.05, a difference was considered significant. Statistical analysis was carried out using SPSS 16.0 program.

## Results

In order to determine the effect and correlation of the dose of NAC, BSO and coenzyme Q_10 _on H_2_O_2 _content variations, we measured the extracellular H_2_O_2 _of maximum, half and quarter-maximum dose on MCF-7 by the DCHF flowcytometric method. The experiment was repeated for different periods of exposure. Results were then compared to the control (null drug) by subtracting the emission of controls (null drug) from the emission of the samples to calculate the variations of H_2_O_2 _content.

To study the effect of different doses of NAC and coenzyme Q_10 _in different time courses on MMP-2 activity of MCF-7 cell line, standard gelatin zymography was performed, and the density of the developed bands were measured and compared with the control sample in each dose and exposure time via post hoc test. Finally, we measured the correlation between H_2_O_2 _content and MMP-2 activity, and drug dose and MMP-2 activity by the Spearman correlation test.

In the case of NAC, correlation was observed between NAC concentration as a thiol antioxidant and the amount of H_2_O_2 _content in different exposure time courses. Flowcytometeric measurements of H_2_O_2 _by emissions of DCHF revealed large correlation (-0.710 in the first 15 hours -0.775 after 24 hours all p < 0.05) with the amount of thiol antioxidant NAC. Results for 6 and 42 hours show a large correlation, however they were not statistically significant.

In 6 and 15 hours of supplementation, mean MMP-2 activity showed significant (p < 0.05) difference in all groups compared to the control group. In full dose NAC vs. control, MMP-2 activity showed a difference of 58.97%, 52.4% and 28.16% in 6, 15 and 42 hours respectively. Coefficents of -0.946, -0.621 and -0.650 correlation coefficient in 6, 15 and 42 hour respectively (p < 0.05) were calculated by correlation analysis. Also a positive medium correlation was observed between content and MMP-2 activity (p < 0.05).

Following Ortega et al experiments on cell lines, in this study, we employed 0.2 mM concentration for BSO to effectively inhibit γ-GCS and to decrease the intracellular content of GSH [30].

The results showed that merely in the first 6 hours of exposure, BSO was effective to increase the content of the cell. At 6 hours, the flowcytomertic analysis showed a positive 0.798 correlation coefficient between H_2_O_2 _increase and dosage (p < 0.05). In these samples, only the concentration of 0.1 mM had a significant increase (20%, p < 0.05) of MMP-2 activity in comparison with control samples by Post Hoc analysis. Although incubation for 6 and 15 hours showed significant increase (p < 0.05) on MMP-2 activity in ANOVA tests, incubation for more than 15 hours had no significant effect of MMP-2 gelatinolytic activity. (Samples incubated for 24 and 42 hours did not show significant dose-responsive correlation with either H_2_O_2 _content or MMP-2 activity.)

### Coenzyme Q_10_

**Figure 1:**
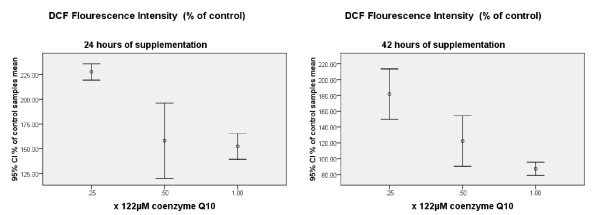
**Measurements of DCF fluorescence as a marker of Hydrogen Peroxide content in the medium after incubating for 24 and 42 hours with serial dilutions of Coenzyme Q_10_**. The numbers represent the percentage of control samples mean.

In this study we used 122 μM of Coenzyme Q_10_, which according to pilot studies had shown no apoptotic or cytotoxic effect on the cells. DCF fluorescence measurements revealed that there is a strong negative correlation between Coenzyme Q_10 _dose and H_2_O_2 _content. Accordingly, 24 and 42 hours of MCF-7 cells exposure to coenzyme Q_10_, illustrated a significant decline of H_2_O_2 _content with a correlation coefficient of -0.926 and -0.739 (p value < 0.05) respectively (Figure 1).

**Figure 2:**
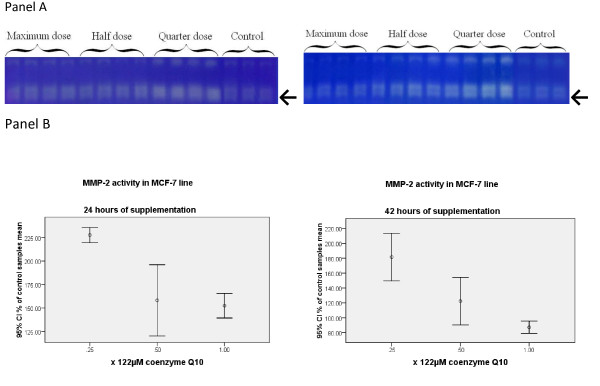
**Panel A. Zymogram charts representing gelatinolytic activity measured by standard gel zymography in 24 and 42 hours of exposure of cells to coenzyme Q_10_**. The arrows point to the row of bands of gelatin digestion. Double bands represent 72 KD MMP-2 and 92 KD proMMP-2. **Panel B**. Statistical analysis presentation of corresponding zymograms: numbers on the y axis represent the percentage of control samples mean.

Samples supplemented merely with Coenzyme Q_10 _for 24 and 42 hours showed significant change in MMP-2 gelatinolytic activity (p < 0.05) (Figure 2). In 24 hours of supplementation, full and quarter dose showed significant (p < 0.05) differences compared to control group mean. Post hoc analysis of samples after 42 hours of incubation revealed significant (p < 0.05) decrease in mean MMP-2 activity when full dose and quarter dose Coenzyme Q_10 _was used. Interestingly, full dose in 42 hours was the only group among Coenzyme Q_10 _supplemented samples that showed less MMP-2 activity compared to control group mean. In correlation analysis, both 24 hours and 42 hours showed large negative correlations (-0.650 and -0.946, p < 0.05) respectively (Figure 2. Panel A).

Finally, statistical analysis of H_2_O_2 _content and MMP-2 activity revealed that there are good correlations (24 hours: 0.772; 42 hours: 0.804, p < 0.05) between H_2_O_2 _content and MMP-2 gelatinolytic activity (Figure 2. Panel B). In other words, this shows that when supplemented with Coenzyme Q_10_, extracellular activity of MMP-2 in MCF-7 cell line is proportionate to H_2_O_2 _concentration.

**Figure 3:**
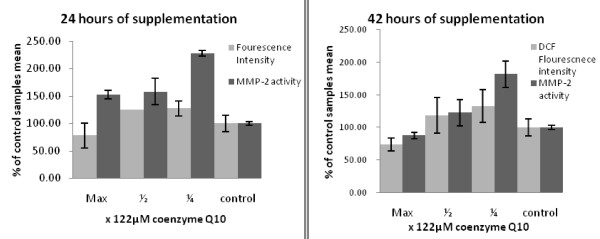
**Illustrations of MMP-2 and DCF fluorescence emission as a marker for H_2_O_2 _content**. Despite similar trends, H_2_O_2 _in both charts is below control levels. However, at 122 μM coenzyme Q_10_, MMP-2 shows lower activity than control only at 42 hours.

After 24 and 42 hours of exposure to Coenzyme Q_10_, both groups at full dose showed significant (p < 0.05) 22 and 24% decrease compared to control group (illustrated in Figure 3).

## Discussion

In the current study, MMP-2 activity as a marker of malignancy was investigated in the presence of various substances that have been shown to play a pivotal role in maintaining the redox status of the cell (namely coenzyme Q_10 _and GSH). Hydrogen peroxide, as an abundant ROS in the cell, was the target of manipulation and measurement.

Nelson et al have carefully reviewed aspects of MMPs activation and regulation by redox status. ROS is considered one of the major routes of MMP regulation through oxidation of the zinc-cysteine switch. It is clear that ROS, particularly H_2_O_2_, play fundamental roles in modulating the various kinase cascades, phosphatase activity, transcription factor binding and MMP latency. Although there are many likely candidates, the specific signaling molecules that are directly modified by ROS and lead to MMP expression have yet to be clearly determined [31].

Sandhya and Mishra had used a concentration of 100 μM for NAC directly on MCF-7 as a suitable non-cytotoxic concentration. In the present experiment, we used serial dilutions of 100 μM to study the dose dependency and the correlation between supplement doses vs. H_2_O_2 _concentration [32].

In almost all exposure times, large correlation was found between NAC and H_2_O_2 _concentrations (15 and 24 hours: p < 0.05, 6 and 42 hours: p > 0.05). ROS was decreased in dose-dependent fashion. This finding was majorly consistent with previous studies. MMP-2 gelatinolytic activity showed a significant (p < 0.05) decrease in all incubation times; and according to our observations this decrease was also in a dose-responsive manner in almost all of the incubation times.

Interestingly, regarding MMP-2 activity change with NAC pretreatment, highest mean difference between groups and the largest correlation (-0.946, p < 0.05) was observed in the first 6 hours of incubation. This observation as well as the large negative correlations between NAC and H_2_O_2 _could indicate that in MCF-7 cell the effect of NAC tends to be more radical scavenging rather than biological inhibiting or inducing. This idea could be supported by the observed positive correlation between H_2_O_2 _and MMP-2 activity (p < 0.05). These findings are partly consistent with reports of Voronkina et al who stated that the effect of NAC on the activities of MMP secreted by normal (3T3) and transformed (3T3-SV40) mouse fibroblasts by supplementation with NAC for 2-6 hours completely inhibited MMP-2 and MMP-9 activity in both cell lines. They speculated that the inhibition was independent of NAC concentration at the range of 1-10 mM [33].

Similarly, the decline of MMP-2 activity in the presence of NAC could be due to the protection of cysteine switch by this monothiol. This postulation is congruent with observations of Bogani et al who used 5 mM NAC [34].

As the most important thiol antioxidant components, glutathione is a tri-peptide involved in converting H_2_O_2 _to water. It functions very closely to NAC which is itself a precursor of glutathione. Its roles have undergone extensive research in areas of prevention and therapy of cancer; it has been shown that MMP-1 mRNA and MMP-2 and -9 activities are inhibited by glutathione in human fibroblasts and liver allografts, respectively. Glutathione has been shown to induce MMP-1 and -2 expressions in human heart fibroblasts. Conversely, in transformed fibroblasts, glutathione inhibited MMP-1 and -2 expressions and increased TIMP-2 expression. Altogether these findings advocate the role of oxidative stress in cancer [35]. N-acetylcysteine has been reportedly able to block the TNF- and high-glucose mediated induction of MMP-9 mRNA and protein as well as the activities of MMP-2 and -9 in human fetal membranes [36].

To investigate its GSH depleting effect on activity/expression of MMP-2, we pretreated cells with BSO (a glutathione synthesis inhibitor). BSO prooxidant behavior was only significant in the first 6 hours of incubation (BSO vs. H_2_O_2_). Also in this period, a significant 20% increase (p < 0.05) of MMP-2 activity was observed. Incubations more than 6 hours did not show any significant change in either H_2_O_2 _or MMP-2 activity. It is possible that after 6 hours, GSH synthesis had resumed by the translation of enzyme γ-glutamylcysteine synthetase or compensation by increasing the rate of antioxidant recycling or other antioxidants synthesis.

Brennisen and Wank et al have proposed that the inhibition of catalase by aminotriazol (ATZ), inhibition of GSHPx by buthionine sulfoximine (BSO), and blocking the Fenton reaction by the iron chelator desferrioxamine (DFO) in concert led to an increase in steady-state MMP-1 mRNA levels, possibly dependent on intracellular H_2_O_2 _increase [37]. In a later study, Wenk et al reported increase in basal MMP-1 expression in consequence of H_2_O_2 _increase as the mediator of cell signaling [38]. From these observations it could be concluded that BSO acts on MMP as a secondary targets via alteration of ROS content of the cell.

Coenzyme Q_10 _has been suggested as the most important antioxidant against H_2_O_2_. Since the cytoplasmic membrane is permeable to H_2_O_2_, it is to be assumed that H_2_O_2 _measured in the microenvironment represents an approximation of intracellular content of H_2_O_2_. In addition, supplemented Coenzyme Q_10 _has been shown to consist a fraction of Mitochondrial Coenzyme Q_10 _[13]. Moreover, Coenzyme Q_10 _action against other elements of acute inflammation has been shown in in-vitro studies [39].

To our knowledge, this is the first time that the effect of Coenzyme Q_10 _on MMP activation/expression has been published, and in this sense, we have found no other parallel experiment to compare our observation.

According to our observations, supplementation with Coenzyme Q_10 _increased the H_2_O_2 _content of cellular environment in 6 and 15 hours of incubations at all doses (data not shown). After 24 and 42 hours of exposure to Coenzyme Q_10_, both groups at full dose showed significant (p < 0.05) 22 and 24% decrease compared to control group (illustrated in Figure 3), respectively. We assume two mechanisms could be accounted for this phenomenon. First, it could be due to oxidative stress caused by high dose exposure of exogenous Coenzyme Q_10_. As indicated previously in this text, Coenzyme Q_10 _freely passes through membranes and, to certain extent, would disrupt the delicately tuned superoxide anion/H_2_O_2 _signaling pathways [40,41] and [42].

Therefore, longer exposures were needed for the cells to adapt to the new redox state. Secondly, it is well known that Coenzyme Q_10 _possesses pro-oxidant properties which, in turn, greatly contribute to sustaining the physiological redox potential of the cytoplasm and membranes. Accordingly, generating the superoxide anion/H_2_O_2 _as second messengers in signaling systems are affected [41]. Our data suggest that pro-oxidant behavior of Coenzyme Q_10 _could have contributed to the increased H_2_O_2 _content observed. It may be possible that in the case of incubation time over 24 hours, enough time was given to the cells to establish a new equilibrium, thus decrease the overall H_2_O_2 _content of the ECM. It is to highlight that due to their molecular properties, both Coenzyme Q_10 _and H_2_O_2 _would face little hindrance for passing the lipid membranes.

In addition to widely accepted effects of H_2_O_2 _on MMP activity, causal role of H_2_O_2 _and quelling role of antioxidants on the expression of MMP mRNAs have been frequently demonstrated in numerous original reports and reviews [37] and [42]. In line with these findings, we assume similar mechanisms may have played a role in the current study. The studied novel modulatory effect of Coenzyme Q_10 _on MMP activity/expression might be mainly due to Coenzyme Q_10 _interaction with ROS mediators. However, further expression studies need to elucidate the exact mechanisms of this interaction.

## Conclusions

Taken together, the present study highlights the significance of Coenzyme Q_10 _effect on the cell invasion/metastasis effecter molecules.

## List of abbreviations

MMP: matrix Metalloproteinase; ROS: reactive Oxygen Species; NAC: N-acetyl cystein; BSO: buthionine sufoximine; DCFH-DA: 2', 9'-dichlorodihydrofluorescein diacetate; TGFβ: transforming growth factor β; HGF: hepatocyte growth factor; TPA: 12-O-tetradecanoylphorbol-13-acetate; MAPK: mitogen-activated protein kinases; PI3K: phosphoinositide 3-kinase; VEGF: vesicular endothelial growth factor; DMEM: dulbecco's minimum essential medium.

## Competing interests

The authors declare that they have no competing interests.

## Authors' contributions

**MB**, **Sh Kh **and **PP**: participated in the study design, carried out the analyses and grafted the Manuscript. **MP**, **MR Kh**, **HM **and **SGN **Participated in study design and coordination and Helped to draft manuscript. All authors read and approved the final manuscript.

## References

[B1] Kessenbrock K Plaks V Werb Z Bicknell R: Matrix Metalloproteinases: regulators of the tumor microenvironment Cell 2010 141 52 67 10.1016/j.cell.2010.03.015 20371345 PMC2862057

[B2] Roy R Yang J Moses MA Matrix metalloproteinases as novel biomarkers and potential therapeutic targets in human cancer J Clin Oncol 2009 27 5287 97 10.1200/JCO.2009.23.5556 19738110 PMC2773480

[B3] Qian Q Wang Q Zhan P Peng L Wei SZ Shi Y The role of matrix metalloproteinase 2 on the survival of patients with non-small cell lung cancer: a systematic review with meta-analysis Cancer Invest 2010 28 661 9 10.3109/07357901003735634 20394501

[B4] Ala-aho R Kähäri VM Collagenases in cancer Biochimie 2005 87 273 86 10.1016/j.biochi.2004.12.009 15781314

[B5] Brown NS Bicknell R Hypoxia and oxidative stress in breast cancer Oxidative stress: its effects on the growth, metastatic potential and response to therapy of breast cancer Breast Cancer Res 2001 3 323 7 10.1186/bcr315 11597322 PMC138696

[B6] Wu WS The signaling mechanism of ROS in tumor progression Cancer Metastasis Rev 2006 25 695 705 10.1007/s10555-006-9037-8 17160708

[B7] Shekelle P Hardy ML Coulter I Udani J Spar M Oda K Effect of the supplemental use of antioxidants vitamin C, vitamin E, and coenzyme Q10 for the prevention and treatment of cancer Evid Rep Technol Assess (Summ) 2003 75 1 3 15523748 PMC4781200

[B8] Yuvaraj S Premkumar VG Vijayasarathy K Gangadaran SG Sachdanandam P Ameliorating effect of coenzyme Q10, riboflavin and niacin in tamoxifen-treated postmenopausal breast cancer patients with special reference to lipids and lipoproteins Clin Biochem 2007 40 623 8 10.1016/j.clinbiochem.2007.02.003 17425952

[B9] Adhikary A Mohanty S Lahiry L Hossain DM Chakraborty S Das T Theaflavins retard human breast cancer cell migration by inhibiting NF-kappaB via p53-ROS cross-talk FEBS Lett 2010 584 7 14 10.1016/j.febslet.2009.10.081 19883646

[B10] Chao HP Kuo CD Chiu JH Fu SL Andrographolide Exhibits Anti-Invasive Activity against Colon Cancer Cells via Inhibition of MMP2 Activity Planta Med 2010 in press 20539971 10.1055/s-0030-1250039

[B11] Bentinger M Brismar K Dallner G The antioxidant role of coenzyme Q Mitochondrion 2007 Suppl S41 50 Epub 2007 Mar 16 10.1016/j.mito.2007.02.006 17482888

[B12] Littarru GP Tiano L Clinical aspects of coenzyme Q10: an update Nutrition 2010 26 250 4 10.1016/j.nut.2009.08.008 19932599

[B13] Portakal O Ozkaya O Erden Inal M Bozan B Kosan M Sayek I coenzyme Q10 concentrations and antioxidant status in tissues of breast cancer patients Clin Biochem 2000 33 279 284 10.1016/S0009-9120(00)00067-9 10936586

[B14] Hertz N Lister RE Improved survival in patients with end-stage cancer treated with coenzyme Q(10) and other antioxidants: a pilot study J Int Med Res 2010 38 293 10.1177/147323000903700634 20146896

[B15] Sachdanandam P Antiangiogenic and hypolipidemic activity of coenzyme Q10 supplementation to breast cancer patients undergoing Tamoxifen therapy Biofactors 2008 32 151 9 10.1002/biof.5520320118 19096111

[B16] Premkumar VG Yuvaraj S Vijayasarathy K Gangadaran SG Sachdanandam P Effect of coenzyme Q10, riboflavin and niacin on serum CEA and CA 15-3 levels in breast cancer patients undergoing tamoxifen therapy Biol Pharm Bull 2007 30 367 70 10.1248/bpb.30.367 17268082

[B17] Premkumar VG Yuvaraj S Sathish S Shanthi P Sachdanandam P Anti-angiogenic potential of CoenzymeQ10, riboflavin and niacin in breast cancer patients undergoing tamoxifen therapy Vascul Pharmacol 2008 48 191 201 10.1016/j.vph.2008.02.003 18407793

[B18] Rusciani L Proietti I Rusciani A Paradisi A Sbordoni G Alfano C Low plasma coenzyme Q10 levels as an independent prognostic factor for melanoma progression J Am Acad Dermatol 2006 54 234 41 10.1016/j.jaad.2005.08.031 16443053

[B19] Ito T Ito M Shiozawa J Naito S Kanematsu T Sekine I Expression of the MMP-1 in human pancreatic carcinoma: relationship with prognostic factor Mod Pathol 1999 12 669 74 10430270

[B20] Cheng CJ Lin YC Tsai MT Chen CS Hsieh MC Chen CL SCUBE2 suppresses breast tumor cell proliferation and confers a favorable prognosis in invasive breast cancer Cancer Res 2009 69 3634 41 10.1158/0008-5472.CAN-08-3615 19369267

[B21] Bogani P Canavesi M Hagen TM Visioli F Bellosta S Thiol supplementation inhibits metalloproteinase activity independent of glutathione status Biochem Biophys Res Commun 2007 363 651 5 10.1016/j.bbrc.2007.09.018 17900531

[B22] Griffith OW Biologic and pharmacologic regulation of mammalian glutathione synthesis Free Radic Biol Med 1999 27 922 35 10.1016/S0891-5849(99)00176-8 10569625

[B23] Pei P Horan MP Hille R Hemann CF Schwendeman SP Mallery SR Reduced nonprotein thiols inhibit activation and function of MMP-9: implications for chemoprevention Free Radic Biol Med 2006 41 1315 24 10.1016/j.freeradbiomed.2006.07.014 17015178 PMC2405910

[B24] Das S Banerji A Frei E Chatterjee A Rapid expression and activation of MMP-2 and MMP-9 upon exposure of human breast cancer cells (MCF-7) to fibronectin in serum free medium Life Sci 2008 82 467 76 Epub 2007 Dec 27 10.1016/j.lfs.2007.12.013 18243246

[B25] Rosenkranz AR Schmaldienst S Stuhlmeier KM Chen W Knapp W Zlabinger GJ A microplate assay for the detection of oxidative products using 2',7'-dichlorofluorescin-diacetate J Immunol Methods 1992 156 39 45 10.1016/0022-1759(92)90008-H 1431161

[B26] Factor VM Kiss A Woitach JT Wirth PJ Thorgeirsson SS Disruption of redox homeostasis in the transforming growth factor-alpha/c-myc transgenic mouse model of accelerated hepatocarcinogenesis J Biol Chem 1998 273 15846 53 10.1074/jbc.273.25.15846 9624185

[B27] Kupai K Szucs G Hajdu I Csonka C Csont T Matrix metalloproteinase activity assays: Importance of zymography J Pharmacol Toxicol Methods 2010 61 205 9 10.1016/j.vascn.2010.02.011 20176119

[B28] Khorramizadeh MR Pezeshki M Ghahary A Zeraati H Berahmeh A Determination of gelatinase A using a modified indirect hemagglutination assay in human prostate cancer screening and assessment of its correlation with prostate-specific antigen parameters Int J Urol 2005 12 637 43 10.1111/j.1442-2042.2005.01094.x 16045556

[B29] Das S Banerji A Frei E Chatterjee A Rapid expression and activation of MMP-2 and MMP-9 upon exposure of human breast cancer cells (MCF-7) to fibronectin in serum free medium Life Sci 2008 82 467 76 10.1016/j.lfs.2007.12.013 18243246

[B30] Ortega A Ferrer P Carretero J Obrador E Asensi M Pellicer JA Down-regulation of glutathione and Bcl-2 synthesis in mouse B16 melanoma cells avoids their survival during interaction with the vascular endothelium J Biol Chem 2003 278 39591 9 10.1074/jbc.M303753200 12881529

[B31] Nelson KK Melendez JA Mitochondrial redox control of matrix metalloproteinases Free Radic Biol Med 2004 37 768 84 10.1016/j.freeradbiomed.2004.06.008 15304253

[B32] Sandhya T Mishra KP Cytotoxic response of breast cancer cell lines, MCF 7 and T 47 D to triphala and its modification by antioxidants Cancer Lett 2006 238 304 13 10.1016/j.canlet.2005.07.013 16135398

[B33] Voronkina IV Kirpichnikova KM Smagina LV Gamaliĭ IA Changes in matrix metalloproteinases activities in normal and transformed mouse fibroblasts under effect of antioxidants Tsitologiia 2008 50 877 81 19062520

[B34] Bogani P Canavesi M Hagen TM Visioli F Bellosta S Thiol supplementation inhibits metalloproteinase activity independent of glutathione status Biochem Biophys Res Commun 2007 363 651 5 10.1016/j.bbrc.2007.09.018 17900531

[B35] Tyagi SC Kumar S Borders S Reduction-oxidation (redox) state regulation of extracellular matrix metalloproteinases and tissue inhibitors in cardiac normal and transformed fibroblast cells J Cell Biochem 1996 61 139 51 10.1002/(SICI)1097-4644(19960401)61:1<139::AID-JCB15>3.0.CO;2-J 8726363

[B36] Lazo JS Kuo SM Woo ES Pitt BR The protein thiol metallothionein as an antioxidant and protectant against antineoplastic drugs Chem Biol Interact 1998 111-112 255 262 10.1016/S0009-2797(97)00165-8 9679559

[B37] Brenneisen P Briviba K Wlaschek M Wenk J Scharffetter-Kochanek K Hydrogen peroxide (H2O2) increases the steady-state mRNA levels of collagenase/MMP-1 in human dermal fibroblasts Free Radic Biol Med 1997 22 515 24 10.1016/S0891-5849(96)00404-2 8981044

[B38] Wenk J Brenneisen P Wlaschek M Poswig A Briviba K Oberley TD Stable overexpression of manganese superoxide dismutase in mitochondria identifies hydrogen peroxide as a major oxidant in the AP-1-mediated induction of matrix-degrading metalloprotease-1 J Biol Chem 1999 274 25869 76 10.1074/jbc.274.36.25869 10464329

[B39] Schmelzer C Lindner I Rimbach G Niklowitz P Menke T Döring F Functions of coenzyme Q10 in inflammation and gene expression Biofactors 2008 32 179 83 10.1002/biof.5520320121 19096114

[B40] Factor VM Kiss A Woitach JT Wirth PJ Thorgeirsson SS Disruption of redox homeostasis in the transforming growth factor-alpha/c-myc transgenic mouse model of accelerated hepatocarcinogenesis J Biol Chem 1998 273 15846 53 10.1074/jbc.273.25.15846 9624185

[B41] Linnane AW Kios M Vitetta L Coenzyme Q10 - its role as a prooxidant in the formation of superoxide anion/hydrogen peroxide and the regulation of the metabolome Mitochondrion 2007 Suppl S51 61 Epub 2007 Mar 30 10.1016/j.mito.2007.03.005 17482887

[B42] Abbasi N Khaghani S Sharif-Tabrizi A Farzami B Vardasbi S Bahar M Comparison of Lipoamide dehydrogenase activity in HL-60 Leukemia cells and normal lymphocyte Acta Medica Iranica 2007 45 282 284

